# A 72 × 60 Angle-Sensitive SPAD Imaging Array for Lens-less FLIM

**DOI:** 10.3390/s16091422

**Published:** 2016-09-02

**Authors:** Changhyuk Lee, Ben Johnson, TaeSung Jung, Alyosha Molnar

**Affiliations:** 1 School of Electrical and Computer Engineering, Cornell University, Ithaca, NY 14853, USA; cl678@cornell.edu (C.L.); bcj25@cornell.edu (B.J.); tj85@cornell.edu (T.J.); 2 Department of Electrical Engineering, Columbia University, New York, NY 10027, USA; 3 Cortera Neurotechnologies, 2150 Shattuck Ave., PH, Berkeley, CA 94704, USA

**Keywords:** CMOS avalanche photodiodes, highly sensitivity photodetectors, fluorescence imaging, photon timing, rangefinder, single photon detectors, SPAD arrays, time-correlated measurements, 3-D image sensor, lifetime microscopy, low power imaging, point-of-care, lab-on-chip, in-vitro, exponential decay, fill-factor, SPAD, photo-detector

## Abstract

We present a 72 × 60, angle-sensitive single photon avalanche diode (A-SPAD) array for lens-less 3D fluorescence lifetime imaging. An A-SPAD pixel consists of (1) a SPAD to provide precise photon arrival time where a time-resolved operation is utilized to avoid stimulus-induced saturation, and (2) integrated diffraction gratings on top of the SPAD to extract incident angles of the incoming light. The combination enables mapping of fluorescent sources with different lifetimes in 3D space down to micrometer scale. Futhermore, the chip presented herein integrates pixel-level counters to reduce output data-rate and to enable a precise timing control. The array is implemented in standard 180 nm complementary metal-oxide-semiconductor (CMOS) technology and characterized without any post-processing.

## 1. Introduction

### 1.1. Overview

Fluorescence imaging has become one of major enabling techniques of modern biology, allowing sub-micron resolution of multiple markers (fluorophores) within a single sample in three dimensions, to monitor dynamic cellular behaviors. Its broader implementation, however, has been limited by fluorescent microscopy’s requirement of bulky optical components, hindering low-cost, miniaturized, and/or implantable imaging systems. Conventionally, resolving fluorophores in 3D requires both (1) high precision scanning and focusing optics and (2) a set of optical filters, such as excitation, dichroic beam splitter, and emission filters to distinguish light emission from different fluorophores and also from (much brighter) stimulus light which are bulky and expensive. In this work, we propose a complementary metal-oxide-semiconductor (CMOS) compatible fluorescence imaging system as a cost effective alternative. We demonstrate a CMOS-based fluorescence lifetime imaging microscopy (FLIM) that is much cheaper and smaller than conventional FLIM systems, at the cost of reduced functional flexibility, sensitivity and 2D spatial resolution. Yet, for many applications such as an in-vivo neural interface, CMOS-based approach possesses several advantages—it can be thinned down (∼10 µm) and made flexible. Furthermore, CMOS can also serve as a versatile platform to incorporate multitudes of modalities to realize a system that can faithfully monitor bio-activities expressed by fluorescence or bio-luminescence [1,2] or by electrical activities [3] with cellular level resolution. To date, lens-less imaging [4,5,6] has been able to expand the field of view without the use of microscope optics, but such efforts require bright-field imaging with structured illumination, which is generally incompatible with fluorescent imaging.

**Figure 1:**
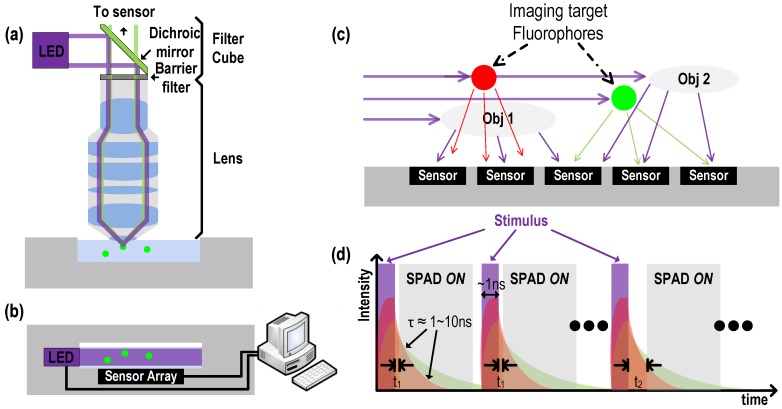
3D imaging without lens and filter (**a**) conventional fluorescent imaging, (**b**) on-chip fluorescent imaging. (**c**) Stimulus light is illuminated in parallel to the sensor surface, the fluorescence emission and scattered from the imaging space (Obj1 and Obj2) reach the sensor surface. (**d**) Time-resolved fluorescent imaging for stimulus rejection and time-gated lifetime detection.

Figure 1a depicts a conventional fluorescent imaging microscope with bulky optical components such as filters and lenses. In this work, we propose the lensless, filterless alternative as depicted in Figure 1b. Such a system not only lowers manufacturing cost by orders of magnitude but also miniaturizes the system while still maintaining core functionality (albeit with reduced resolution and signal-to-noise ratio (SNR)). This approach is amenable to a wide range of applications poorly suited to conventional FLIM systems, including: implantable applications where small size and weight are critical, surgical and endoscopic instruments, cost-sensitive field deployments, and massively parallel assays.

The largest and most expensive components in the aforementioned conventional fluorescent imaging setup are the stimulus light, optical filters, and lens. Stimulus light cannot be eliminated because it is the source of excitation energy; however, many efficient solutions have been proposed to reduce its cost and size, such as the use of a µLED [7] or an on-chip optical waveguides that couples solid-state excitation through the chip from a shared off-chip source [8].

### 1.2. Replacing Filters: Time-Gated SPAD Drive

In all cases, the excitation light source has orders of magnitude higher power (>5 mW/mm^2^) than the fluorescence emission of interest (power ∼10 nW/mm^2^), which makes it essential for the sensor to be able to significantly reject the excitation signal. The geometry by which stimulus light is projected onto a sample can also be arranged parallel to the sensor surface to minimize direct illumination of the sensor. But, as shown in Figure 1c, even when the stimulus field is aligned so as to minimally irradiate the sensor array, scattering of excitation by the sample medium itself may cause saturation due to the finite dynamic range of the sensor.

Optical band-pass/notch filters in the range of the excitation wavelength are widely used for this purpose. For an on-chip imaging configuration, however, interference-based filters are too thick, and provided limited rejection to wide-angle illumination from scattering, while relatively thin (∼30 µm) absorption-based filters have an insufficient rejection ratio of ∼30 dB [9]. Thus, an alternative rejection technique is required that is more compatible with an integrated CMOS solution. An alternative to wavelength specific filters is to use a synchronized pulsed stimulus and high speed solid state circuitry to time-gate sensing to times when only fluorescent emission is present: fluorophores decay from an excited to ground state with a half-life on the order of nanoseconds, meaning that significant numbers of fluorescent photons may be detected after the end of a brief stimulus pulse as depicted in Figure 1d. Single photon avalanche diodes (SPADs) co-integrated with high speed CMOS electronics are ideally suited to this functionality.

### 1.3. Fluorescence Lifetime Imaging

In order to distinguish between fluorescent markers, or detect dynamic changes in their fluorescence due to physiological signals such as calcium concentration, one must resolve changes in the intensity, wavelength and/or temporal statistics of fluorescent emission. Intensity based fluorescent imaging of physiological signals uses changes in fluorescent emission intensity to detect changes in biological state or events in the interior of cells. Unfortunately, accurate measurement of fluorescent emission intensity can be quite challenging due to randomness of a probe concentration and changes in light path loss in dynamic, scattering medium. Wavelength-based approaches, and especially ratio-metric wavelength measurements normalize these effects, providing much better results by providing the proportion of fluorophores in a given state (i.e., bound vs. unbound to calcium). Such approaches, however, require multiple, closely spaced wavelength filters. Like a wavelength reporter, a fluorescent lifetime reporter is independent of the probe intensity, but instead of the wavelength of emission, lifetime imaging is sensitive to the nanosecond-scale changes in emission intensity after excitation. As with wavelength measures, lifetime measurement enable quantitative ratio-metric measurements of fluorophore state, but rely on high-speed sensors and circuits rather than selective optical filters. Extensive search for lifetime probes is on-going and expanding to enable sensing of changes in Ca^2+^, Mg^2+^, K^+^ concentration as well as pH [10,11,12]. In FLIM, fluorophores are distinguished by their decay constants (*τ*’s) rather than by their wavelengths (color) or absolute intensity. Previous work has demonstrated FLIM with arrays of SPADs in standard CMOS with standard microscope optics [13,14]. Lens-less FLIM with SPAD arrays has also been demonstrated [15], but it did not have the ability to localize fluorophores in 3D space and had limited 3D resolution for objects not in direct contact with chip surface.

### 1.4. Replacing Lenses: Angle Sensitive Pixels

Due to the absence of collimation of imaged light, spatial resolution when imaging without focusing optics is inherently inferior to that possible with a conventional focusing system. Nevertheless, significant progress has been made on bright field lens-less imaging, where two techniques are most common. The first is *contact mode imaging* which minimizes light spreading between imaged object and sensor array by placing them close together and collimating the illumination. To compensate for finite pixel size, this method repetitively scans the imaging object while shifting either position of the imaging object relative to the sensor, and/or shifts the position/direction of the light source relative to the object [5] which also can provide parallax for 3-D imaging. The second approach utilizes partially coherent illumination and measures interference patterns between non-scattered and scattered illumination through the imaging object [16]. Unfortunately, neither of these techniques are suitable for on-chip volumetric fluorescence lifetime imaging, where all detected light is emitted isotropically.

**Figure 2:**
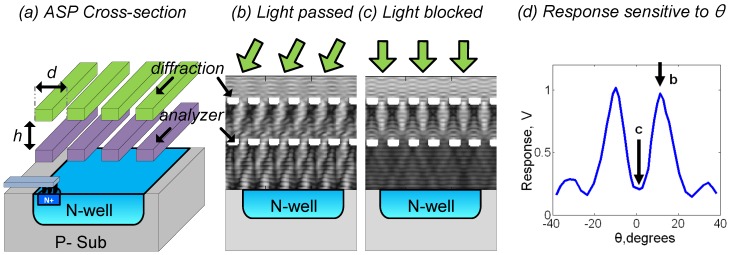
(**a**) Structure of simple angle sensitive pixel (ASP) [17] showing metal gratings and photodiode, grating pitch, ***d*** is order 1 µm, while vertical separation, ***h*** is order 3 µm, (**b**,**c**) finite-difference time-domain (FDTD) simulation of field intensity in response to incident plane waves of light: (**b**) diffraction pattern from top grating aligns with gaps in bottom grating, light passes, (**c**) pattern aligns with bars, light is blocked. (**d**) simulated intensity at detector as angle is swept. FDTD simulation is performed using MATLAB. Inter connect metal gratings (Al) is modeled as perfect electrical conductor (PEC) and the inter dielectric layer (${\mathrm{SiO}}_{2}$) is assumed lossless. The incident angle of the plane wave are swept.

One way to resolve this drawback is through lens-less light field imaging, where the image sensors resolves incoming light rays in both space and incident angle. Light-field imagers have shown promising results by utilizing computational re-focusing [17], lens-less far-field imaging [18], and on-chip imaging [19]. Among approaches to light-field capture, angle-sensitive pixels (ASP) provide an easily integrated, effective solution by extracting angle information about the rays arriving at each pixel. ASPs uses a set of two µm-scale gratings to create an incident-angle-dependent diffraction patterns and then filtering the light based on the offset of this pattern, as shown in Figure 2 [17]. The grating structure is compatible with a standard CMOS process and does not require any post-processing steps such as mounting micro-lenses/lens on the image sensor surface which are required for a light-field imager [20].

### 1.5. Angle Sensitive Time Resolved Fluorescence Lifetime Imaging

This work combines area-efficient, time-gated SPADs with CMOS-compatible integrated optical structures, similar to those used in ASPs [17,18,19] to replace conventional optical filters and lenses. The 2-D incident angle information not only improves non-contact 2-D spatial resolution of light sources above the plane of the chip but also expands the system’s imaging resolution into 3D for volumetric localization. Here we present a 72 × 60, angle-sensitive SPAD (A-SPAD) array fabricated in a conventional 180 nm CMOS process. The pixels temporally reject high-powered UV stimulus pulses [21] while successfully performing 3D localization of different fluorescent sources with different lifetimes through reconstruction utilizing angle information and lifetime measurements, all without the use of lenses or wavelength filters.

In the next section (Section 2) we describe the design of the angle-sensitive SPAD image sensor. Section 3 provides a system-level overview of the architecture of the image sensor and associated challenges. Section 4 shows experimental results demonstrating the function of our image sensor and supporting circuitry. Finally, Section 5 describes the 3D reconstruction algorithm tailored to the unique challenges associated with lens-less 3D FLIM imaging and summarize our work.

## 2. Angle-Sensitive Single Photon Avalanche Diode

### 2.1. A-SPAD Pixel Structure and Circuitry

**Figure 3:**
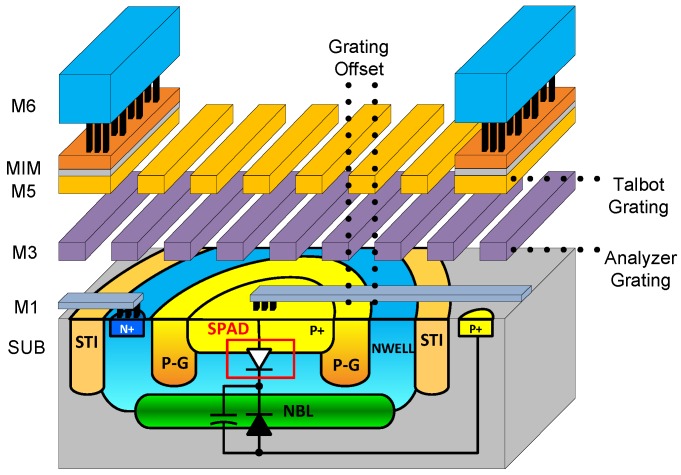
A cross-section of the proposed angle-sensitive single photon avalanche diode (A-SPAD) structure. Reproduced from [22], with the permission of AIP Publishing.

Figure 3 shows a cross-section of A-SPAD structure. The SPAD is formed by the junction between the P+ implant and N-Well with P-epi layer used as a guard ring [13,21]. The two sets of metal gratings for ASP are implemented using conventional CMOS metal stacks, arranged to modulate light based on azimuth and altitude incident angles (*θ* and *φ*) [17]. As light strikes the top grating, an incident angle dependent diffraction pattern is generated beneath it, similar to what is shown in Figure 2b,c. The lower, analyzer grating, then selectively blocks or passes the generated diffraction pattern depending on relative lateral position. The response of an angle-sensitive pixel to light of intensity $I_{0}$ and incident angle *θ* is proportional to $I_{0}\cdot \left(1+m\cos\left(\beta \theta +\alpha \right)\right)$, where modulation depth *m*, angular frequency *β*, and angle offset *α* are design parameters dictated by the geometry of the gratings (***d*** and ***h*** in Figure 2). In order to provide maximum 3D information, our array uses six types of A-SPAD pixels, resulting from combination of two angular frequencies (*β* = 8, 15) [22] and three phases (*α* = −180°, −60°, 60°). Note that prior work in ASPs used four phases (differential sampling of I and Q: *α* = 0°, 90° 180°, 270°), but only resolved three parameters describing incident angle distribution: sinusoidal phase and amplitude, and a background offset, thus conventional four phase sampling has one phase redundancy. To remove the redundancy and increase the spatial sampling efficiency we used three phase sampling where instead of merely removing single phase from the quadrature sampling all three samplings are evenly distributed in phase (*α* = −180°, −60°, 60°) which simplifies the amplitude computation: a numerical average of three output. Both the horizontal and vertical grating orientation are required to make the A-SPAD array sensitive to angle in both *x*-*z* or *y*-*z* planes, *θ* and *φ* respectively. Thus the overall array is made up of tiles of 12 A-SPADs each.

**Figure 4:**
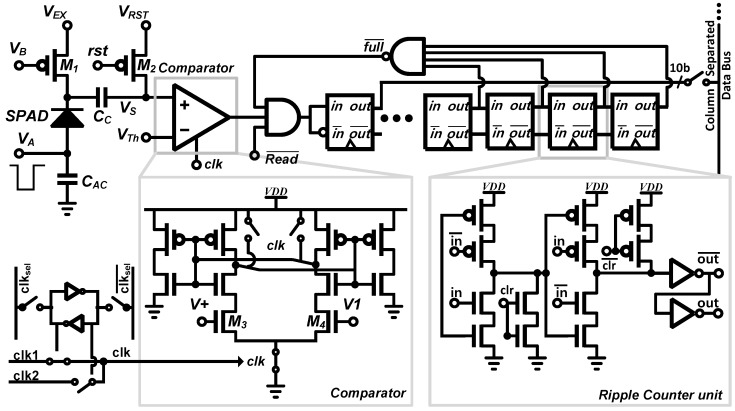
Pixel level schematic of A-SPAD.

Figure 4 shows the schematic of a single A-SPAD pixel with circuitry for time-gated passive quenching (PQ) using transistor $M_{1}$, an AC-coupled comparator to detect photon arrival, and a 10-bit ripple counter for Time Correlated Single Photon Counting (TCSPC). For the time-gated operation, every SPAD’s anode is connected to a global off-chip driver, $V_{A}$, which drives the SPADs beyond their breakdown voltage ($V_{BD}$) into Geiger mode (ON) by the excess voltage ($V_{E}=V_{A}-V_{BD}$). $V_{A}$ is synchronized to the timing of the stimulus light source such that the SPAD remains insensitive ($V_{A}<V_{BD}$) until after UV excitation, when it is turned *"ON"*. Detection of a photon results in a step voltage drop across the SPAD by $V_{EX}$ which couples through a MIM capacitor ($C_{C}$) to the $V_{S}$ node, on the input of the comparator.

### 2.2. Readout Circuitry: Comparator and Counter

The sensitivity of the SPAD is increased as the reverse bias rises above the ($V_{BD}$), as is dark count rate. Thus, the globally controlled reverse bias needs to be adjusted in order to regulate and optimize array-wide photon counting to avoid desensitization and saturation. A fixed voltage pulse detector such as an inverter [13,21] has a lower limit on pulse amplitude detection when the supply voltage is fixed, and lowering supply voltage degrades temporal precision in the detection. Therefore a differential comparator, rather than an inverter, was implemented for pulse detection in this work. The schematic of the comparator is shown in Figure 4. The comparator’s detection threshold ($V_{Th}$) can be adjusted to track the coupled voltage step on $V_{S}$ which is linearly dependent on the excess bias voltage ($V_{E}$). The freedom to control threshold voltage also allows compensation of the process, voltage, and temperature (PVT) variation in SPAD breakdown voltage ($V_{BD}$). The device size of the input pair in the comparator is designed to minimize DC offset($\sigma_{V_{th}}$ < 4.76 mV) and node capacitance to minimize detection uncertainty and quenching current. Furthermore, a positive feedback (latch) load with reset switch provides high gain to reduce timing uncertainty.

Data readout of SPAD array can be approached in several ways: (1) a single-bit readout can be stored in a single bit memory in pixel [21,23], (2) a single bit multiplexed to a shared off-pixel time interval counter (TDC) [24] or (3) a multi-bit type that uses an in-pixel TDC [14], (4) a multi-bit counter/analog integrator that accumulate photon detection events. The first type is efficient for a small scale arrays and provides good fill factor but requires all SPADs be read out after every excitation pulses limiting frame rate as the number of pixels increases. The second type achieves similar fill factor, and much better single-frame time resolution, but can only capture from a small subset of SPADs at once, requiring many excitation pulses to capture a full frame. The third approach allows simultaneous reading from many SPADs, but at a cost in fill factor, and still requires high data bandwidth to sustain steady frame rate [25]. The last approach, which is implemented in the proposed imager, has a reduced data bandwidth thanks to an in-pixel counter/integrator however at the cost of reduced fill factor [26,27].

The data bandwidth is especially critical in a moderate or low figure imaging environment, since acquiring an accurate time-histogram requires averaging over a large number of measurements. The minimum number of photon detections necessary for fluorescence lifetime measurements (monoexponential decay analysis) can be derived [28], and a typical estimation of fluorescence lifetime histogram requires averaging over 500 photon detection events [24]. At the same time, the average photon detection activity must be controlled to be on the order of 1%∼5% to avoid non-linearity due to photon pile-up [29] or local saturation/desensitization. This means that with 1% average photon detection activity, over 50,000 measurements are required to produce a single lifetime histogram [24]. If such TDC measurements were to be exported off-chip for a large scale array, the data bandwidth requirement can easily become impractical. For instance, full histogram reconstruction of a 72 × 60 system with 10 bit TDC and 10ns laser repetition rate, at worst case, would require an approximate bandwidth of 540 GB/s (=10×72×60/10 ns/byte). This is even more true for A-SPADs, since meaningful angular information (which is derived from diffraction, and so the wave-description of light) can only be extracted by measuring the average of discrete photon detection events (derived from the particle description of light) [22]. On-chip data compression or decimation may partly resolve this issue (event-driven readout [30], embedded FIFO [31]), but still suffers from inefficiencies due to moving inherently redundant data from the array core to the compression circuitry [24]. For the A-SPAD array, described here, which operates in a low figure environment while collecting extra dimensions of information, we took the simple yet efficient alternative approach of pixel level averaging combined with tightly controlled detection windows. This allows the read-out rate from each pixel to be reduced by the number of measurements being averaged, while increasing the number of bits to be read out by log_2_ (♯ measurements × $P_{activity}$) where $P_{activity}$ (a probability of positive output) is limited to ∼1% to avoid pileup. For example, with 10 bit in-pixel accumulator and ∼1% activity rate we can increase the number of averaged measurement up to 100 ksamples before the in-pixel memory over flows.

**Figure 5:**
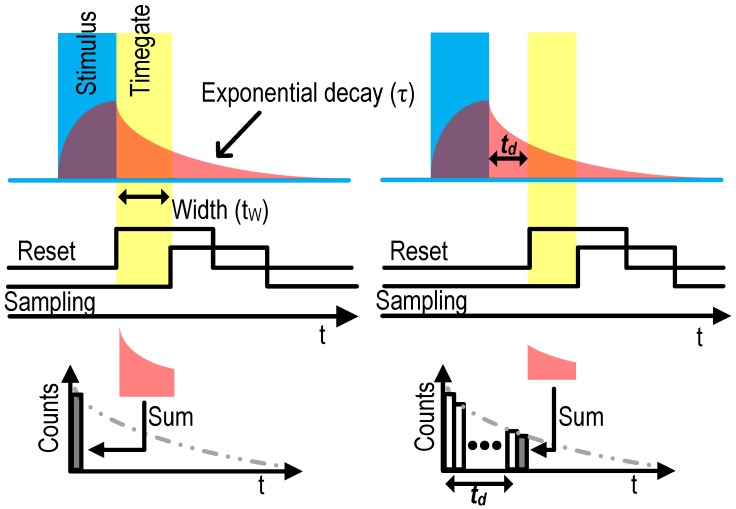
Statistical sampling with detection window width ($t_{W}$), detection window shift ($t_{d}$), lifetime (*τ*) for lifetime estimation. Note that the proposed system is able to achieve $t_{W}$ = $t_{d}\geq 72$ ps whereas, $t_{W}∼2.4\times \tau$ is used in this work.

To increase the frame rate and provide sufficient figures with pixel level averaging, a 10 bit asynchronous ripple counter was used to integrate output of the comparator over multiple excitation pulses. As shown in Figure 5, the counter consists of tri-state inverters with cascaded asynchronous reset switches and output buffers. Using a fixed window accumulation method significantly reduced the overall data rate compared to the TDC method, albeit requiring more total frames (as much as a factor of TDC bit resolution when time-gate does not overlap) to generate a histogram with equivalent SNR (discussed in Section 3).

**Figure 6:**
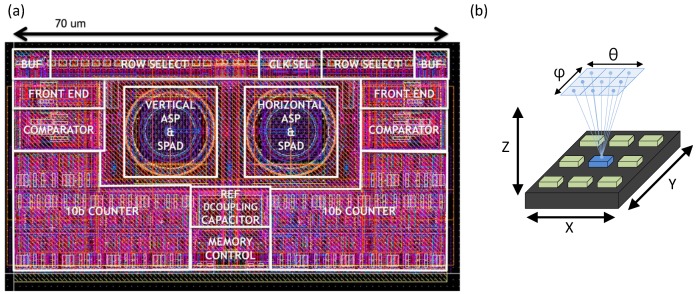
(**a**) Layout of two (*θ* and *φ* sensitive) A-SPAD pixels. (**b**) illustration of *θ* and *φ*.

A layout of two A-SPAD pixels (*θ* and *φ* sensitive) are shown in Figure 6. A fill factor of 14.4% (15 µm N-well diameter) was achieved through a simple pixel-circuit design, which is dominated by a 10 bit ripple counter and overlaid MIM capacitors which double as a light shield.

## 3. Large Scale A-SPAD Array Design

### 3.1. Power and Area Efficient Lifetime Estimation Approach

FLIM depends upon the ability to resolve fine time differences in photon arrival time statistics (≤1 ns). Conventional methods for lifetime estimation can be classified into two categories: (1) time gating (TG) method using a digital counter [32,33,34,35], and (2) TCSPC method using time-to-digital converter (TDC) [14,24,25,36].

Time-gating methods are efficient when a large number of samples are needed to provide *"analog like"* intensity information [37]. When using time-gating methods, algorithms such as rapid lifetime determination (RLD) and its variants such as overlap RLD (ORLD) and center-of-mass method (CMM) based RLD are widely used for their simplicity. Unfortunately, this method extracts the average lifetime of multi-exponential decays when they coexist, introducing fixed pattern noise/extension to the estimation [38], and the gate position and interval have to be adjusted based on assumption of lifetime. The TCSPC method combined with an in-pixel TDC (minimum of eight time bins [38]) is close to ideal for producing an unbiased histogram, but comes at the cost of drastic increase in hardware complexity, area, power consumption and high I/O data bandwidth for off chip curve fitting.

A real-time time gating method requires a minimum of two, four and eight bins to estimate single-exponential, bi-exponential and multi-exponential decays, respectively. Although the multi-exponential decay time constant can be extract by small number (eight) of time bin, high temporal resolution raw histogram data is often preferred [35]. For the proposed design, we use a hybrid of the two methods. We use a time gating method for its simplicity and compactness of circuitry, but introduce a dynamically programmable time shift on the gate, with fine temporal accuracy (∼100 ps), to enable sweeping of the time-gate, enabling extraction of multi-exponent demixing. Specifically, we repeat the laser excitation and measurement cycles many times for a given gating time offset, counting the photon detection within the time-gate. Lifetime estimation is acquired by shifting the time-gate with fine temporal resolution (>72 ps) relative to the laser pulse. Here the resulting photon count creates an average sum of inhomogeneous Poisson random variables over the gating time. The accumulated count within the detection window can be interpreted as an integration of the combined probability density functions of all light sources illuminating the SPAD (See Figure 5). The time-gate is formed with three global timing references generated by on chip digital-to-time converters (DTC). Short time-gate width ($t_{w}$) can provide simple and intuitive histogram for lifetime estimation where the proposed system can reduce the width as short as DTC LSB (>72 ps); however, increases required number of stimulus and sampling to achieve reasonable SNR. Thus, instead of using the minimum width (LSB), we have used a 10 ns time-gate width analogues to the optimal time-gate width of the RLD method for τ≥4 ns where $t_{w}\geq$= 2.4×τ [35]. The start of the detection window is applied to the gate of the p-FET (M1) reset switch. When it is high, the positive input of the comparator ($V_{S}$) sees a high impedance and follows the AC coupled avalanche induced voltage drop from the SPAD. The end of the time-gate is defined by the comparator clock.

### 3.2. Digital-to-Time Converter (DTC)

The fine resolution time-gate shifting and counting can benefit from a multi-channel synchronous clock reference on chip. For continuous imaging, we used a rolling shutter based technique to read out *"analog like"* accumulated digital counts from each pixel. In a conventional rolling shutter, each sensor’s data accumulation occurs synchronously with a global time-gated detection window, and the number of photons counted per pixel can be controlled by the number of cycles between memory reset and read. The time histogram, as shown earlier in Figure 5, can be acquired by using a sliding time-gated window. Efficiently combining a sliding window with rolling shutter requires pixel-by-pixel recording and readout, where each pixel, as it is read out and reset, is switched from one timing window to the next. This requires two precisely controlled windows, which are generated using digital-to-time converters (DTC).

**Figure 7:**
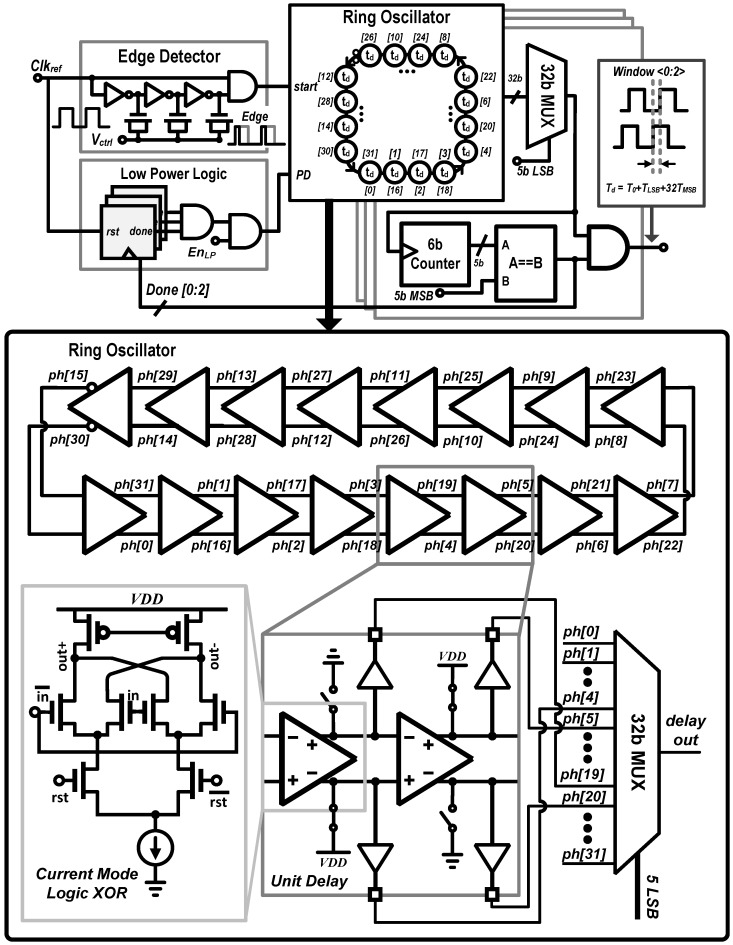
Schematic of high dynamic range, area efficient DTC, where *ph[0:31]* are nodes between each delay unit, the supply voltage *VDD* is 1.8 V, *rst* denotes reset signal to stop the ring oscillator (RO), *Done[0:2]* is a digital bit to power gate the RO to enable the low power DTC operation.

Figure 7 shows the schematic of the DTC used in this work. For minimal area and optimal power efficiency, the open-loop delay-line utilizes a ring oscillator instead of a long single line or a binary weighted programmable delay. For a synchronous open-loop operation, a single reference clock is provided from off-chip. Both positive and negative edges of the reference clock are extracted by positive/negative edge detector which initiates a positive/negative edge ring oscillator whose output is connected to three delay lines to set the relative timing between the excitation pulse and the two time-gates for rolling shutter operation. Each delay line has a coarse control (5-MSBs) that selects the number of cycles around the oscillator. The oscillator consist of 16 differential delay units, and nodes between each delay unit are connected to a thermometer-coded multiplexer which feeds each counter to provide 5 LSBs of finer control. Each delay unit is a current mode differential exclusive or (XOR) logic gate whose tail current can be adjusted to control the LSB delay (<80 ps). To counter the effects of changing the tail current, the p-FET biasing is maintained by an off-chip negative feedback loop. This structure allows flexible and accurate control of both duration and delay of the detection window relative to the reference. Furthermore, to conserve power, it is possible to power down the ring oscillator after all three time-gates are generated by putting it in a low power ($En_{LP}$) mode.

Time-gates generated by the DTC, in conjunction with an in-pixel clock pointer and a single bit static random-access memory (SRAM) to select the on-going detection window, are used to implement the rolling shutter scheme. A pixel readout is followed by thecounter memory reset.

### 3.3. System Architecture

**Figure 8:**
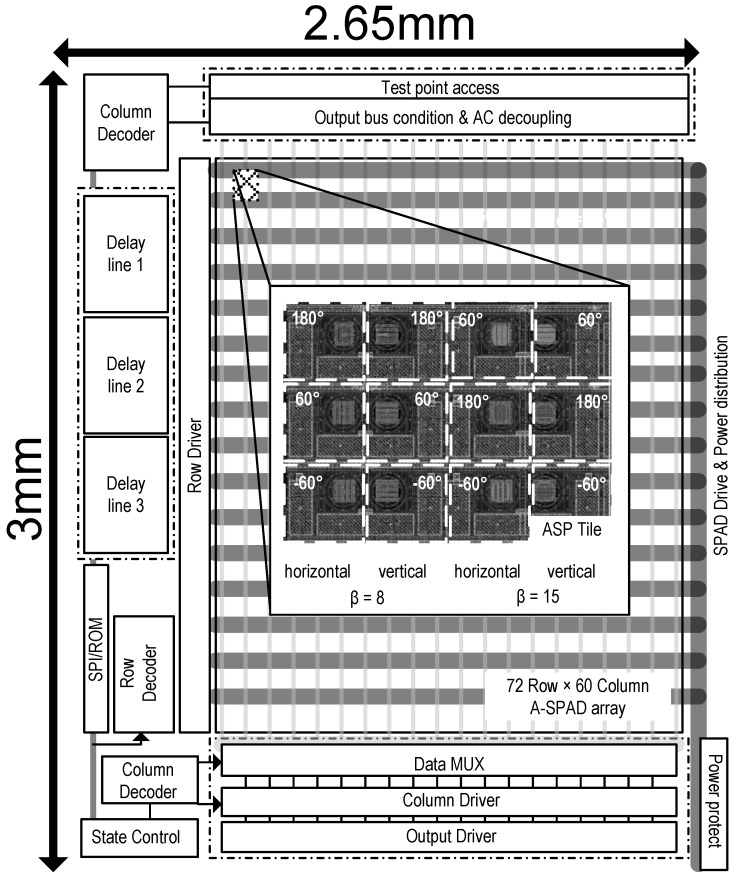
System architecture of a 72 × 60 A-SPAD array.

Figure 8 shows the architecture of imager system. The system synchronizes an off-chip UV stimulus to the internal delay lines which in turn generate detection windows. Other supporting circuits around the A-SPAD imaging core include a column and row decoder for pixel access, data MUX for a readout, SPI for serial communication to off-chip FPGA, the two DTCs with three outputs, and a system state machine.

This simple system architecture is robust; during three-months of continuous testing, the system reliably operated without a system reset.

The Photon detection probability (PDP) for an A-SPAD (with Talbot and analyzer gratings) is 2.72% at 540 nm with $V_{EX}$ = 1.2 V whereas SPAD without gratings resulted in PDP of 18.7 % indicating a factor of 7 loss due to the reflection from gratings [22]. The array wide (72 × 60 pixels) median dark count rate across the array was 404 Hz [22]. Including local the comparator and the 10 bit ripple counter, the pixel pitch is 35 µm × 35 µm. The chip achieves a fill-factor of 14.4% across the active area of the array, and 9.6% including support circuits and pads. The system consumes 73.8 mW or 83.8 mW power in dark or tested illumination conditions respectively.

The sensitivity, or detection efficiency, is one of the photon detector’s (SPAD) most important performance factors. Numerous studies have worked to enhance the quantum efficiency or/and fill factor of SPADs [39]. However, in many cases, the sensitivity measurement is performed at the single device level and underestimates the various challenges that may arise in a large scale array. A notable example is fluctuations in local sensitivity caused by the large avalanche current pulse of adjacent pixels, which is a binary response containing very sharp edges. To put a SPAD into Geiger mode (ON), its reverse bias (≥ 10 V) is supplied from off-chip due to the complexity and limitations of generating such high voltage using standard CMOS process. Because the various interconnects, including wire-bond ($L_{bond}$) and on-chip routing, introduce parasitic resistance and inductance, they present high impedance to the high frequency components of the avalanche current and cause local voltage drops. Despite the use of wide traces for current return path and multiple wire-bonds (6 total) to stabilize $V_{A}$, avalanche events inevitably cause local and global fluctuations. Since reducing $V_{A}$ reduces sensitivity these fluctuations in $V_{A}$ modulate pixel sensitivity across the array and degrade the overall image quality.

**Figure 9:**
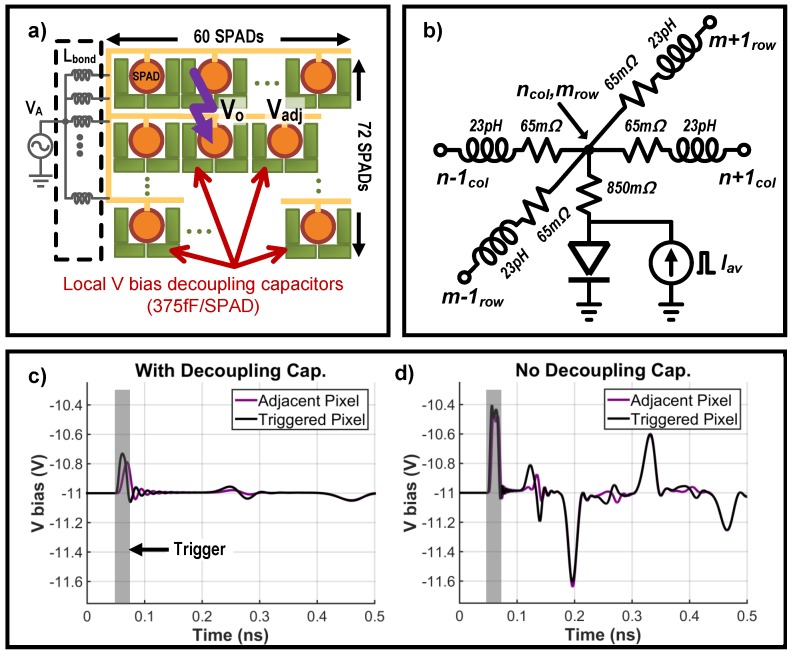
(**a**) Schematic of SPAD reverse bias network with local decoupling capacitors, (**b**) SPAD bias network impedance model. (**c**) Simulated SPAD reverse bias voltage with decoupling capacitor (**d**) Simulated SPAD reverse bias voltage without decoupling capacitors where red and black lines depict avalanche pixel and neighboring pixel respectively.

To evaluate the effects of internal bias line impedance on the avalanche count of a single pixel, an array scale simulation illustrated in Figure 9a was performed using a simple lumped pixel model in Figure 9b. The integrated SPAD array may experience global transient drops in bias voltage when one or more SPADs are triggered in a short time window. The combined avalanche current through finite bond-wire impedance lowers $V_{A}$, reducing sensitivity and limiting the number of photons that can be detected in a short period of time. Simulation result of the reverse bias $V_{A}$ variation is depicted in Figure 9c. The local voltage drop from the avalanche current couples to the reverse bias of adjacent SPAD pixels, desensitizing them, and so distorting or even saturating their response. To mitigate this effect, several steps were taken; six parallel wire-bonds that supply $V_{A}$ were interleaved with current return path bonds to minimize effective inductance, and each pixel contained a decoupling capacitor ($C_{AC}=375$ fF/SPAD) to absorb a portion of the avalanche transient current. As a result, the amplitude of the local $V_{A}$ drop was reduced by a factor of 2.5 as shown in Figure 9c. The decoupling capacitor (two series connected MIM) and AC coupling MIM cap $C_{C}$ also serve as optical shield for active circuitry against any undesirable photo-current generation, especially suppressing leakage from dynamic nodes in the in-pixel counter.

## 4. Results

### 4.1. Measurement Setup

**Figure 10:**
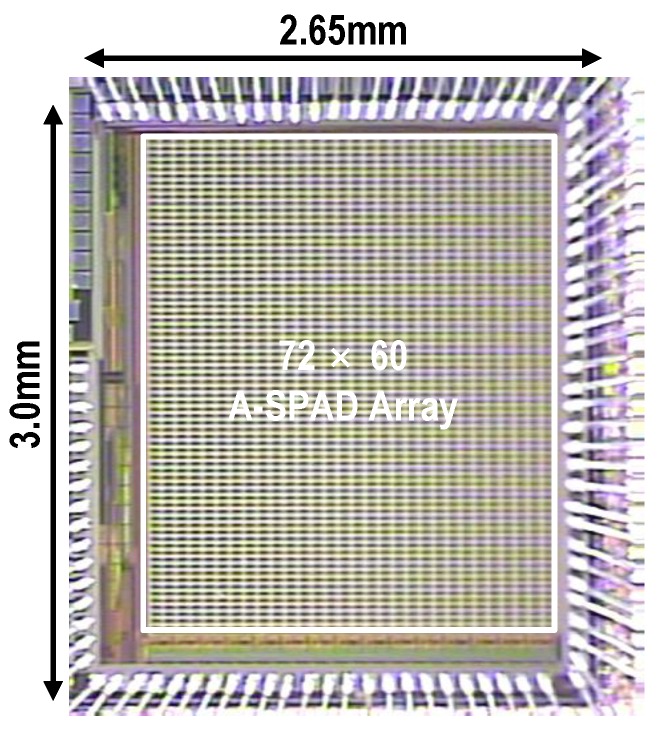
Micro photograph of the A-SPAD FLIM IC.

Figure 10 shows the micro-photograph of the A-SPAD FLIM integrated circuit (IC), fabricated in a standard 180 nm CMOS process. The IC was interfaced with a field-programmable gate array (FPGA) (Cyclone II, Altera, San Jose, CA, USA) for serial peripheral interface (SPI) control, and 20 low-voltage differential signaling (LVDS) (2 × 10 bit) output channels were connected to a high speed digital data acquisition module (NI PXIe-6555, National Instruments, Austin, TX, USA). The lifetime histogram and image reconstruction were post-processed in MATLAB.

### 4.2. Angular Sensitivity Response

**Figure 11:**
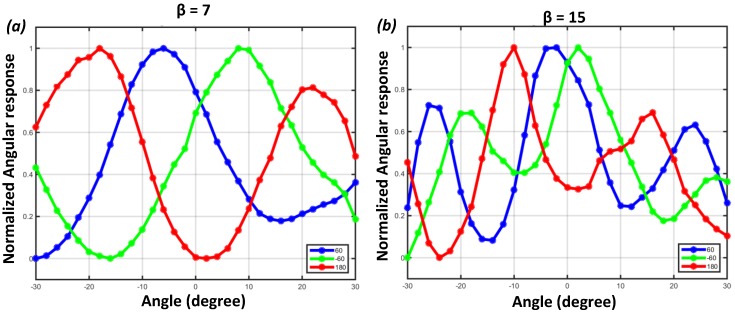
Measured A-SPAD responses to change in incident angle. (**a**) β=8; (**b**) β=15.

A-SPAD’s response to change in incident angle (*θ*, *φ*) was measured by rotating the sensor surface relative to a collimated beam of light generated by green LED (*λ* = 540 nm). Figure 11 shows measured angle response curves for all six A-SPAD pixel types. Again, this is a combination of three different phases (α=60°,180°,−60°) and two spatial frequencies (β=8,15) where β=8 in Figure 11a refers to low frequency (sparse grating) and β=15 in Figure 11b refers to high frequency (dense grating) pixels. The agreement between measurement (accumulation of single/few photon measurements) and simulation in Figure 2 where binary SPAD readout represents particle-like behavior of light and the FDTD simulation represents wave-like behavior of light complies with the particle/wave duality of light and verifies that A-SPAD is capable of detecting angle dimensions, making its sensing dimension a total of 5: *X*, *Y*, *φ*, *θ*, and time [22].

### 4.3. Digital to Time Converter (DTC) Performance

**Figure 12:**
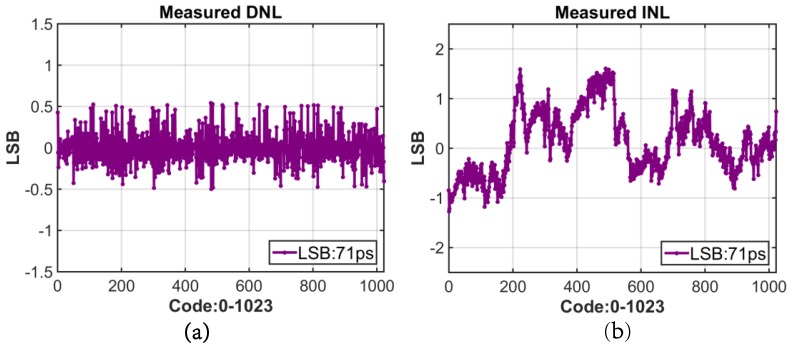
(**a**) DNL of the DTC; (**b**) INL of the DTC.

Static performance was measured for each of the three global DTCs. They were independently provided with a 20 MHz external reference clock (equivalent to highest laser repetition rate), and the delay between input reference and DTC output was measured with a 350 MHz Universal Counter/Timer (Agilent 53230A, Agilent Technologies, Santa Clara, CA, USA ). The outputs of the DTC were connected to test mode I/O pads to characterize the linearity (integral nonlinearity (INL) and differential nonlinearity (DNL)). Ten measurements were collected and averaged for each delay code. The LSB delay can be adjusted by controlling the tail current of the XOR delay unit shown in Figure 7. The change of voltage swing of the current mode logic (CML) due to change in tail current was compensated by an off-chip voltage regulator [40] and pFET load. The CML voltage swing was set to 1.6V minimize jitter and the LSB delay to 72 ps. Figure 12a,b show the resulting DNL and INL of the DTC. The maximum measured DNL is less than 0.54 LSB, and INL is less than 1.27 LSB. The measured high jump around the MSB switch of INL is similar to our layout extracted simulation results, which implies that this abruptness may have been caused by the parasitic capacitance in layout routing. The root-mean-square (RMS) jitter averaged over the full dynamic range was 13.2 ps, and power consumption was 24.1 mW with 1.8 V supply voltage.

## 5. 3D Localization Using FLIM

### 5.1. Measurement Setup

**Figure 13:**
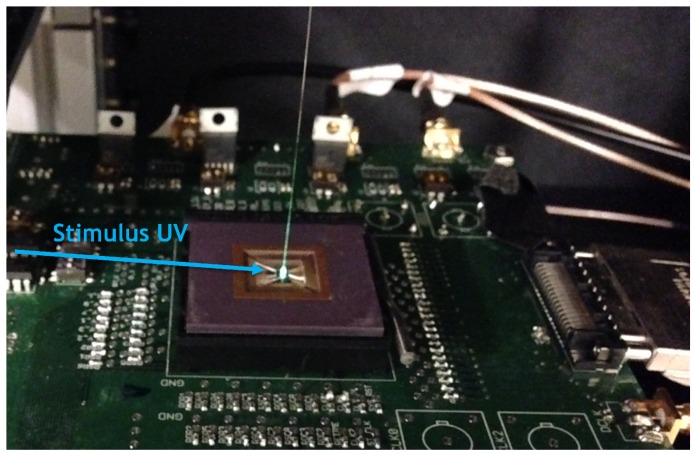
Measurement setup for on-chip 3D localization and fluorescence lifetime extraction.

Two sets of measurement were taken: 3D localization based on angle sensitivity and lifetime information, and fluorescence lifetime extraction. Figure 13 shows the setup for both measurements. For each measurement, images were taken with two fluorescent micro-spheres suspended above the A-SPAD array. For 3D localization, two types of solid fluorophore grains—Muscimol Bodipy TMR-X conjugate and Fluoresbrite YG—with diameters around 100 µm were used. For fluorescence lifetime measurement, two types of quantum dot (QPP-450 and QSP-560, OceanNanotech, San Diego, CA, USA) were cured in epoxy to emulate/encapsulate imaging targets.

The imaging targets were made as small as possible to generate response close to point spread functions. For each case, the two fluorescent beads were positioned above the A-SPAD array using a micro-manipulator. The stimulus light was provided by a UV (*λ* = 385 nm) laser diode (DeltaDiode, HORIBA Scientific, Kyoto, Japan) with an average optical power of 5 mW/mm^2^. For each case, the fluorophores were stimulated by 50 ps wide pulses at a rate of 2 MHz by UV beam parallel to the plate.

### 5.2. 3D Localization Based on Angle Sensitivity and Lifetime Information

**Figure 14:**
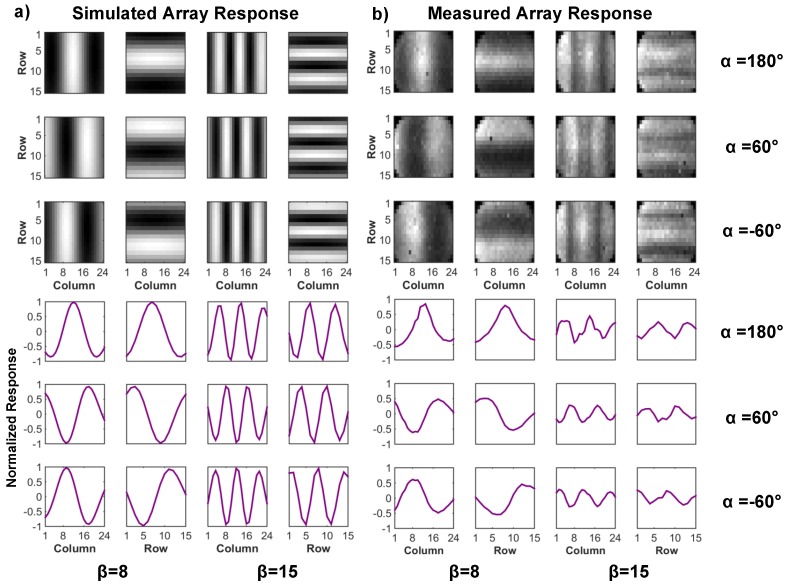
(**a**) Ideal array-wide angular response to a point source with computed *A* matrix; (**b**) Actual angular response to a point-like light source.Note: Ideal angular response is derived from $I_{out}\left(\theta \right)\;=\;I_{o}\left(1+m\;cos\left(\beta \theta +\alpha \right)\right)\times F\left(\theta \right)$, where m = 1 [22].

Prior to performing 3D localization, the array-wide angular response to a point light source was verified. This response was first computed considering the total 12 configuration (six pixel types in *X*-*Y* alignment. See Figure 8) per tile of the A-SPAD array. To measure the actual response, an LED light passing through a pinhole was placed far away from the array to generate a beam from a point light source. This beam was passed through a high aperture lens placed on top of the array so that the resulting light spread would look like it originated from a point source. To extract an angular response, average intensity data of three different phase samplings, is subtracted from each individual pixel response. Figure 14 shows the comparison between computed and measured angular response of the array.

Localization was performed based on the array-wide angular response, with intensity information (from the average response of ASPs with different angular sensitivities) normalized out. This did not degrade the accuracy of 3D localization suggesting that there is more information in incident angle than intensity data. Furthermore, suppressing intensity information provides better tolerance against measurement artifacts such as fixed pattern noise.

**Figure 15:**
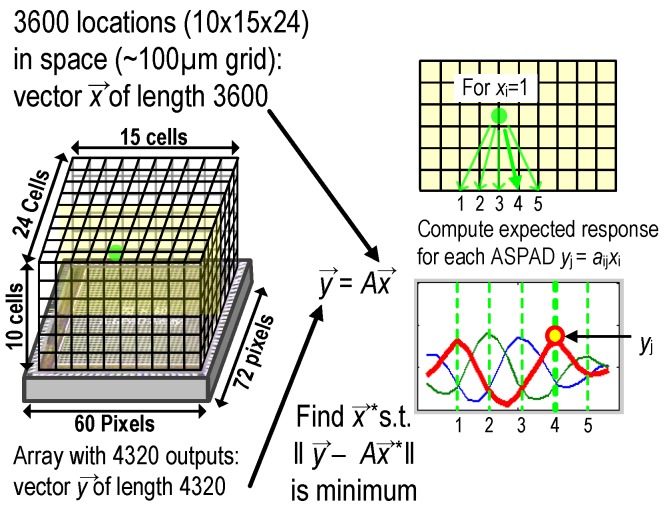
Illustration of 3D localization method based on angular response.

3D localization algorithm was implemented by extracting the sparse set of source locations ($\vec{x}$) that best explain the A-SPAD outputs ($\vec{y}$). When an output of the array $\vec{y}$ and its back-projected location $\vec{x}$ have matched dimensions, *A* becomes an invertible matrix, leading to a definitive localized solution $\vec{x}=A^{-1}\vec{y}$. However, due to the dimension mismatch between two vectors $\vec{x}$ and $\vec{y}$, ($\vec{x}$ describes a volume whereas $\vec{y}$ denotes a plane) the mapping matrix *A* is not square and therefore not invertible. Fortunately, localizing fluorophore in 3D can be formulated as a quadratic program ($∥\vec{y}-A\vec{x}∥$) when the number of fluorophore clusters are limited to a low number (i.e., they are sparse). Figure 15 illustrates the overall process as well as specific dimensions of $\vec{x}$, $\vec{y}$, and *A*.

To be more specific, our localization algorithm made use of pseudo-matrix A^+^, combined with iterative search similar to the bootstrapping method. Starting with initial response $\vec{y}$, likely positions of fluorophores, ${\vec{x}}^{*}$, were found with back-projection $A^{+}\vec{y}$ and thresholding. Then, ${\vec{x}}^{*}$ was multiplied with *A* to generate a simulated response ${\vec{y}}^{*}=A{\vec{x}}^{*}$ where entries in $\vec{x}*$ that lead to a large discrepancy between ${\vec{y}}^{*}$ and $\vec{y}$ were discarded. Finally, ${\vec{y}}^{*}$ was used to re-start the reconstruction cycle by making a second guess on likely positions of fluorophores. Such iterations eventually lead to a converged solution that reconstructs the fluorophore positions. In addition, we have controlled the fluorophore movements with micro-manipulators, which of resulting movement vectors are then compared to the reconstructed images to confirm the validity of estimation.

Compared to lensed systems, the proposed system here suffers from reduced lateral resolution: a lensed system is limited to a lateral resolution set by pixel size and magnification, and or by the diffraction limit of the light itself (100s of nm). The lensless approach described here, does not benefit from magnification, and so for objects close to the chip is limited by the pixel pitch itself (35 µm). For more distant objects, this resolution is limited by the effective angular resolution of the A-SPADS, which is, in the angle domain, $\delta x,y=\frac{\pi }{\beta_{MAX}}$ and translates to $\frac{2\pi \times z}{\beta_{MAX}}$ in the spatial domain, where z is the vertical distance from the chip to the plane being resolved. Vertical resolution is this same term, scaled by the cosine of the angular aperture ($\theta_{app}$) of the A-SPADs: $\delta z=\delta x,y\times cos(\theta_{app}/2)$. Compared to a typical lensless contact imager, this resolution is improved by a factor of $\frac{2\times \pi }{\theta_{app}}$. Thus, resolution wise, the proposed approach falls between full, high resolution lensed systems, and typical lens-less systems. While a quantitative study to analyze key metrics of the reconstruction such as spatial resolution, estimation error, robustness, convergence rate, effect of scattering, number of sources and computational overhead in a greater detail is certainly needed to better understand the system and such performance parameter is inevitably a strong function of algorithms which of details will merit a paper of its own [41].

**Figure 16:**
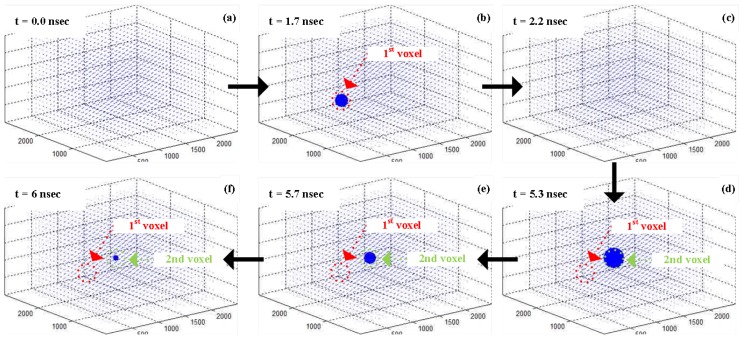
Example snap-shot frames for reconstructing the fluorophores.

**Figure 17:**
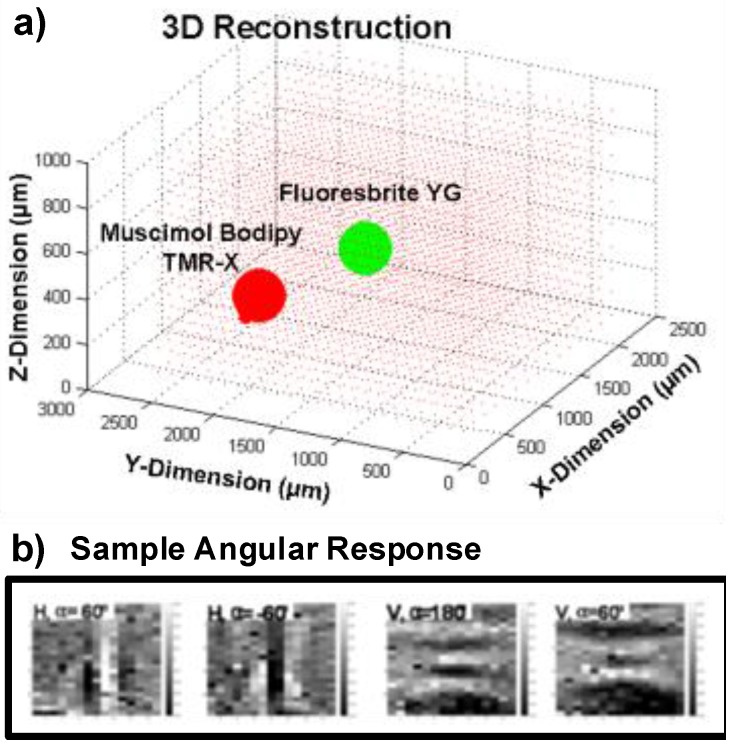
(**a**) 3D localization of two types of fluorophore micro-spheres. (**b**) Sample angular response from these micro-spheres.

For this measurement, a 10 ns wide time-gate was shifted in steps of 139 ps, corresponding to two LSB bits of DTC, with 3D localization performed for each time step. The distinctive lifetimes of the two fluorophores—Muscimol Bodipy TMR-X (red) and Fluoresbrite YG (green)—make the solution sparser and easier to reconstruct. The reconstructed peak location for each shifted time-gate are shown in Figure 16. The distinct temporal responses of the two voxels whose response was above threshold reconstruction correspond to each lifetime of the two fluorophores. The global sensitivity which is a function of the SPAD reverse bias was adjusted to meet the SNR requirements to localize the Muscimol Bodipy TMR-X conjugate (Red voxel) due to the poor absorption efficiency at single photon excitation wavelength (385 nm). As a result, the laser power and the array sensitivity was excessive for the Fluoresbrite YG microspheres (Green voxel), resulting in partially saturated response from many SPADs avalanching at once due to photons from the Fluoresbrite YG. This can be alleviated by simply adding an additional excitation source where each of fluorophore types can be more efficiently stimulated. Nevertheless, even with single excitation source a 3D location of two distinct fluorophore voxels are accurately reconstructed as shown in Figure 17.

### 5.3. Fluorescence Lifetime Measurement

**Figure 18:**
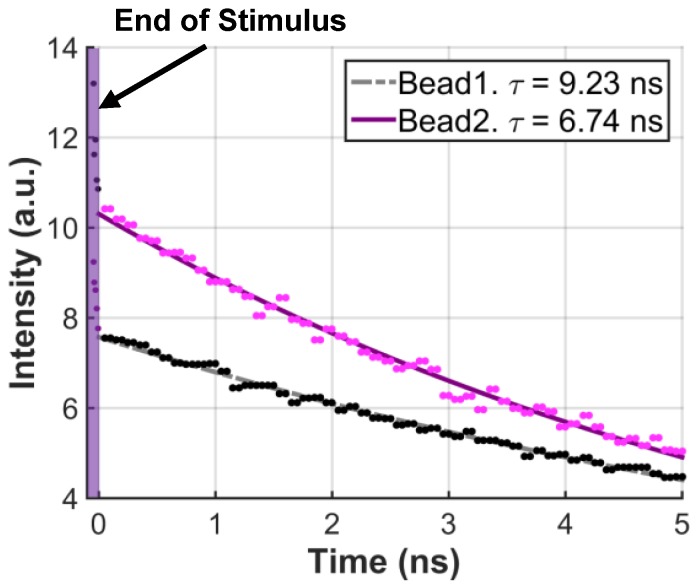
Lifetime extraction for quantum dots (Bead1: QSP-560, Bead2: QPP-450).

The two fluorophores used above exist as fine solid grains in µm scale and were thus suitable for 3D localization. Because their excitation wavelength is far away from UV regime, however, it was difficult to obtain sufficient intensity data for their lifetime extraction. To properly test the DTC’s capability to resolve shorter lifetimes, two quantum dots whose lifetimes are in scale of nanosecond were measured. Because quantum dots are not functional in solid state, however, they were first dissolved in toluene and then mixed with curable, transparent epoxy (Vitralit 1688, Epoxy Technology, Billerica, MA, USA) to form minuscule beads. These beads, however, were difficult to localize in 3D because they were significantly bigger than solid fluorophore grains, which violated sparsity condition. Furthermore, epoxy’s uneven surface caused irregularities in photons’ incident angles on the pixel surface. Quantum dot lifetimes alone, however, could be clearly distinguished despite the small difference ∼2.5 ns between them. Figure 18 shows the resulting lifetime curves extracted for each quantum dot. Intensity data was collected by shifting the 10 ns wide detection window by 71 ps, by averaging three measurements per time bin. The collected histogram data was curve-fitted to produce lifetimes of 9.23 and 6.74 ns for QPP-450 and QSP-560, respectively.

The first measurement shows that with combination of lifetime signature and the pre-localized 3D position, the system can localize distinct fluorescent sources in 3D without any lens or filter. The second measurement shows that with the control of detection window formulated by the system’s DTC combined with in-pixel averaging, it is possible to resolve nanosecond fluorescence lifetimes with low off-chip data bandwidth.

## 6. Conclusions

Apart from the powerful conventional fluorescence microscopy, there still is a significant need for low cost and compact imagers that can potentially become implantable devices for in vivo biological applications in the future. Heading towards that goal, in this paper we have built a first reported 3D on-chip fluorescence lifetime imaging system without the use of any lenses or filters both of which are critical to the conventional FLIM system. To eliminate those components, we used a CMOS compatible optical structure, an angle-sensitive pixel, to extract angle information for localization of fluorophores without lens, and circuit techniques to generate accurately controllable detection window and perform on-chip data compression to obtain fluorescence lifetime histogram without any filter. In essence, we have combined SPAD’s high sensitivity with metal gratings much like the recent works in angle sensitive pixel (ASP), to make an image sensor closer to the ideal 8D plenoptic function sensor [42]. The simple system architecture is optimized for robustness and high fill-factor with uniform sensitivity across the array of 4230 A-SPAD pixels. To generate global timing window for photon detection, a 10 bit DTC with LSB of 71 ps, RMS jitter of 13.2 ps, and 24 mW power consumption was used. The total power consumption of the IC is 83.8 mW. Other performance highlights are summarized in Table 1.

## Figures and Tables

**Table 1:** Comparison with other state-of-the-art SPAD FLIM image sensors.

	This Work	[14]	[43]	[24]	[44]	[45]
PDP (Max %) @Ve	2.72 ^†^@1.2 Ve	25@1 Ve	20@3 Ve	30@1.5 Ve	-	40@5 Ve
Pixel Size	35 µm × 35 µm	50 µm × 50 µm	25 µm × 25 µm	48 µm × 48 µm	8 µm × 8 µm	100 µm × 100 µm
Fill Factor	14.4%	2%	4.5%	0.77%	19.63%	3.14%
DCR (kHz)	0.404@1.2 V*_E_*	0.1@1 V*_E_*	0.078@3 V*_E_*	0.54@2.5 V*_E_*	0.05@2 V*_E_*	4@5 V*_E_*
Array Format	72×60	32× 32	128 × 128	64 × 64	256 × 256	32 × 32
Chip size	2.64 mm × 3 mm	4.8 mm × 3.2 mm	25 mm × 25 mm	9.1 mm × 4.2 mm	3.5 mm × 3.1 mm	3.5 mm × 3.5 mm
Power	83.8 mW	90 mW	363 mW	26.4 W	-	-
Process	180 nm	CIS 130 nm	350 nm	130 nm	CIS 130 nm	350 nm
**Lifetime Method**	**Time-Gated (DTC)**	**TDC**	**Time-Gated**	**TDC**	**Time-Gated (TAC)**	**Time-Gated**
Resolution (LSB)	<71 ps	119 ps	200 ps	62.5 ps	6.66 ps	-
Max Range	72.7 ns	100 ns	9.6 ns	64 ns	50 ns	-
DNL (LSB)	0.54	0.4	-	4	3.5	-
INL (LSB)	1.27	1.2	-	8	-	-
**Application**	**3D FLIM**	**FLIM**	**FLIM**	**FLIM**	**FLIM**	**FLIM**
Lens	No	Yes	Yes	-	Yes	Yes
Filter	No	Yes	No	Yes	-	Yes

† includes losses due to ASP gratings. *V_E_*: excess voltage. *TAC*: time-to-amplitude converter. *TDC*: time-to-digital converter. *DTC*: digital-to-time converter.

## Abbreviations

SPAD	Single Photon Avalanche Diode
FLIM	Fluorescence Lifetime Imaging Microscopy
CMOS	Complementary metal-oxide-semiconductor
TCSPC	Time Correlated Single Photon Counting
SRAM	Static random-access memory
